# Relationship between animal-based on-farm indicators and meat inspection data in pigs

**DOI:** 10.1186/s40813-024-00359-9

**Published:** 2024-01-25

**Authors:** Johanna Witt, Joachim Krieter, Kathrin Büttner, Thore Wilder, Mario Hasler, Ralf Bussemas, Stephanie Witten, Irena Czycholl

**Affiliations:** 1 Institute of Animal Breeding and Husbandry, Christian-Albrechts-University, 24098 Kiel, Germany; 2 Unit for Biomathematics and Data Processing, Faculty of Veterinary Medicine, Justus Liebig University, 35392 Giessen, Germany; 3 Lehrfach Variationsstatistik, Christian-Albrechts-University, 24118 Kiel, Germany; 4 Johann Heinrich von Thünen Institute, 23847 Westerau, Germany; 5 Department of Veterinary and Animal Sciences, University of Copenhagen, 106 91 Frederiksberg, Denmark

**Keywords:** Animal-based indicators, Animal welfare, Animal health, Animal welfare assessment, Slaughterhouse indicators, Slaughterhouse findings, Meat inspection data, Pig

## Abstract

**Background:**

This study aimed to validate slaughterhouse indicators collected during meat inspection as an alternative to on-farm animal welfare indicators. For this purpose, the assessments of twelve on-farm and seven slaughterhouse indicators of 628 pigs from three different farms were combined into three indices, differentiated between on-farm and slaughterhouse: (1) limb health, (2) other organ health, and (3) respiratory health. At first, an assessment at animal-level using agreement parameters was carried out to ascertain whether the same welfare or health issues were identified on-farm and at slaughterhouse, taking the production period (farrowing, rearing and fattening period) and the last weeks before slaughtering into account. Second, the connection of slaughterhouse findings on the individual on-farm health indices was examined using logistic regressions, to determine whether certain welfare issues can be better monitored using slaughterhouse indicators.

**Results:**

Acceptable agreement was determined using the Prevalence-Adjusted Bias-Adjusted Kappa (PABAK) for the farrowing and fattening period, but not for the rearing period. A more detailed analysis of the weeks before slaughter shows that there is still a poor agreement 8 weeks before slaughter and an acceptable agreement 4 weeks before slaughter. This indicated the slaughterhouse indicators pneumonia, pleuritis and pericarditis as possible estimators of fever and deviant behavior on-farm and the slaughterhouse indicators bursitis and joint inflammations as possible estimators of lameness. In the second part of the analysis, the connection of slaughterhouse findings on the individual on-farm health indices was investigated; a significant influence of the farm on the limb and respiratory indices and no significant influence of the slaughterhouse findings could be determined, provided that all weekly assessments during the lifetime of the pigs have been taken into account. However, an influence of the slaughterhouse findings on the respiratory index and on the other organ index could be determined if only the weekly assessments four and eight weeks before slaughter, respectively, were taken into account.

**Conclusions:**

In general, the possible suitable indicators detected by the PABAK, could replace some health-related indicators but a complete substitution of on-farm welfare assessment is not possible. In addition, the traceability over time must be investigated further.

## Background

In the last two decades, the health and welfare of farm animals has increasingly become the focus of society [1]. To address its concerns and to protect animals, the European Union (EU) has implemented a number of regulations. Therefore, minimum standards for the protection of pigs on farms, during transport and at the slaughterhouse have been stipulated. Among other things, the use of animal-based indicators to monitor animal welfare on-farm was recommended [2, 3]. Following on from the ‘Welfare Quality Assessment Protocol for Pigs’, which evaluates animal welfare on-farm mainly using animal-based indicators [4]. The advantage of animal-based indicators is that they best describe how the animal copes with the current environment [5]. However, some animal-based indicators show weaknesses in reliability and in general; the feasibility of on-farm assessments is questionable due to time requirements [6]. Thus, a general interest in the outcomes of meat inspection data from the slaughterhouse as indicators of a retrospective on-farm animal welfare and health monitoring tool has arisen [7, 8]. The meat inspection at the slaughterhouse has the main function of ensuring food safety and is EU legislation, thus it is required for pigs slaughtered for meat [9]. Based on this legislation, the framework additionally enables the recording of slaughterhouse indicators that could be related to animal welfare. The use of such animal-related indicators (such as lesions) at the slaughterhouse and using them to establish a relation to animal welfare on the farm is also recommended by the European Food Safety Authority [10, 11]. Consequently, slaughterhouses become a bottleneck in the food production chain and can provide important information regarding health. In addition, meat inspection achieves a significantly higher level of feasibility, as a large number of pigs can be assessed in a short period of time in comparison to on-farm assessments [12]. It has been possible to validate some slaughterhouse indicators already, for example tail and skin lesions, and classify them as ‘iceberg indicators’, which are indicators with a high prevalence and indicate more than one welfare problem [11, 13]. It is still questionable to what extent early stages of production can be controlled by these indicators and whether the origin of e.g. lesions can be correctly assigned [12, 14].

Therefore, the study aimed to validate slaughterhouse indicators collected during meat inspection as an alternative to on-farm welfare indicators. Selected on-farm welfare indicators were assessed on pigs and slaughterhouse indicators of these pigs were recorded additionally. These data were used to determine the potential substitution of some on-farm indicators by slaughterhouse indicators in order to increase the feasibility of animal welfare assessments.

## Results

### Prevalence on-farm/slaughterhouse indicators

**Fig. 1:**
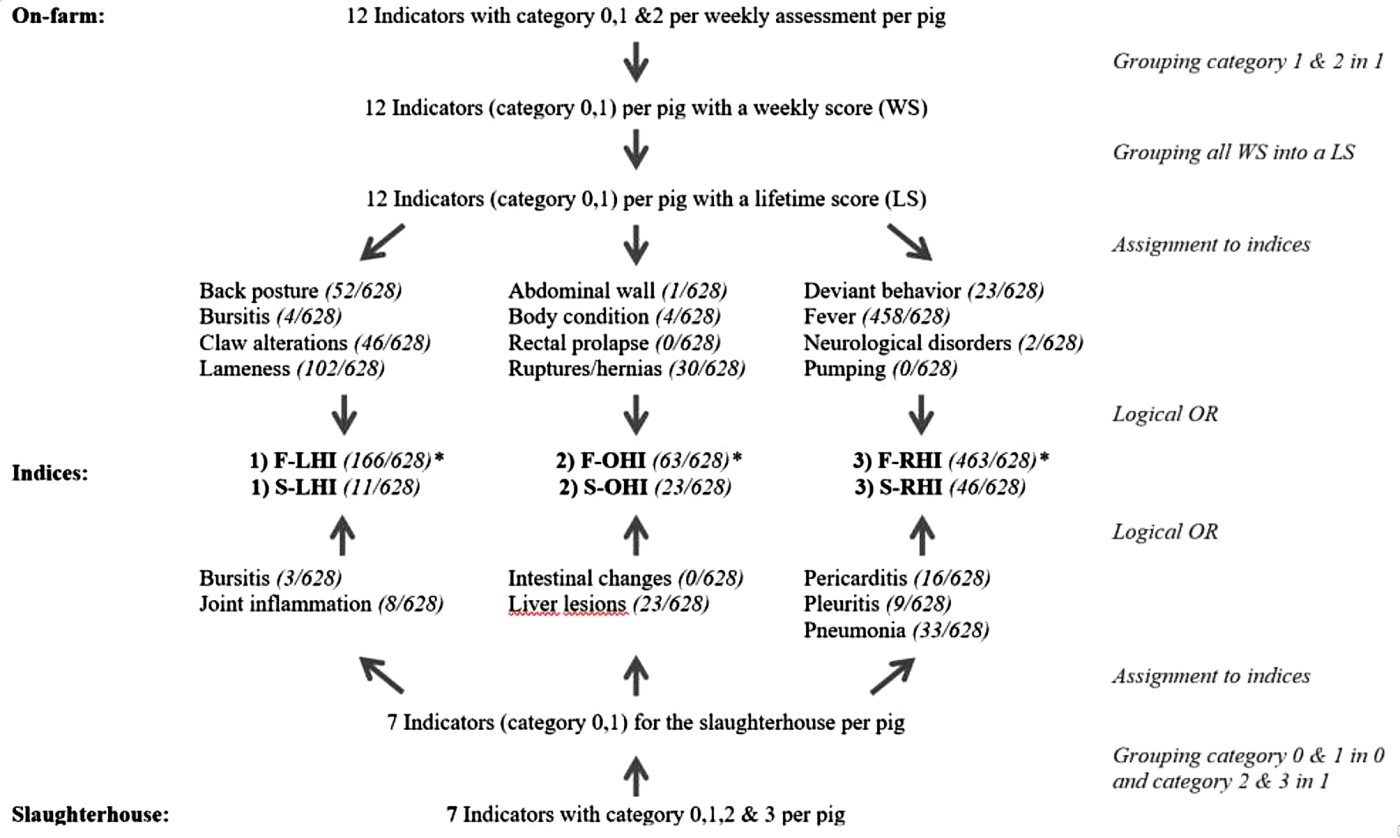
Aggregation of the on-farm/slaughterhouse indicators (number of affected (= 1) pigs of all pigs (*n* = 628) in the study) to create the lifetime health indices (LHI = limb health index; OHI = other organ health index; RHI = respiratory health index) per pig on-farm (F) and at the slaughterhouse (S) with the logical OR-operator, (QS, 2020); ^*^Not identical with the sum of the number of affected animals of the individual indicators, as animals can present with more than in one indicator

As shown in Fig. 1, of the on-farm indicators, fever affected the highest number of pigs (458 pigs; 72.9%), followed by lameness (102 pigs, 16.2%) out of 628 pigs. The other indicators ranged between 52 pigs (8.3%) for back posture and 0 pigs (rectal prolapse, pumping). Among slaughterhouse indicators, out of 628 pigs, 33 pigs (5.3%) were affected by pneumonia, followed by liver lesions (23 pigs; 3.7%). The remaining indicators ranged from 16 affected pigs for pericarditis to 0 pigs (intestinal changes), representing less than 3.0%.

### Prevalence on-farm/slaughterhouse health indices

Of a total of 628 pigs, 166 pigs (26.4%) were assessed on-farm as affected (1 = present) for the on-farm limb lifetime health index (F-LHI), 63 pigs (10.0%) for the on-farm other organs lifetime health index (F-OHI), and 463 pigs (73.7%) for the on-farm respiratory lifetime health index (F-RHI). The corresponding 95%-confidence intervals (CI) were 9.35 to 23.9 for the F-LHI, 3.32 to 9.28 for the F-OHI, and 29.81 to 62.79 for the F-RHI. For the slaughterhouse indices, eleven pigs (1.8%) for the slaughterhouse limb health index (S-LHI), 23 pigs (3.7%) for the slaughterhouse other organs health index (S-OHI) and 46 pigs (7.3%) for the slaughterhouse respiratory health index (S-RHI) scored 1 (= present) out of 628 pigs (see Fig. 1). The corresponding 95%-CI for these slaughterhouse indices were 0.00 to 2.24 for the S-LHI, 0.14 to 4.46 for the S-OHI, and 1.90 to 7.30 for the S-RHI.

### Agreement parameters

The calculated agreement parameters between the equivalent on-farm and slaughterhouse indices for different production stages (time periods) are presented in Table 1. Considering only the assessments (assessment 1 to assessment 4) during the farrowing period, an acceptable agreement for both the OHI and the LHI was achieved by the percent agreement (PA) (0.94; 0.94) and by the prevalence-adjusted bias-adjusted kappa (PABAK) (0.53; 0.53). A good agreement for the RHI was determined using the PABAK (0.60). In the rearing period (assessment 5 to assessment 12) for the OHI and the LHI a good agreement was calculated by the PA (0.95; 0.96), but for no other parameter. No acceptable or good agreement could be achieved for the RHI in this production stage. Considering the fattening phase, values of acceptable agreement for PA and PABAK were calculated for all three indices, but not for Cohen’s Kappa Coefficient (*k*) (see Table 1). A detailed analysis of the last weeks before slaughter shows that eight weeks before slaughter (8weeks) there is still a low agreement and four weeks before slaughter (4weeks) an acceptable agreement.

**Table 1:** Percent agreement (PA), Cohen’s kappa coefficient (*k*) and prevalence-adjusted bias-adjusted Kappa (PABAK) indicating poor (normal type), acceptable (italic type) and good agreement (bold type) for the comparisons of the on-farm and slaughterhouse indices for the different production stages (assessments (a) used in brackets) and four (4weeks) or eight (8weeks) before slaughtering

Production stage	Limb health			Other organ health			Respiratory health		
	PA	k	PABAK	PA	k	PABAK	PA	k	PABAK
Farrowing (a1-a4)	*0.94*	0.02	*0.53*	*0.94*	0.02	*0.53*	0.80	0.02	**0.60**
Rearing (a5-a12)	**0.96**	0.06	0.05	**0.95**	0.01	0.06	0.85	0.05	0.15
Fattening (a13-a30)	*0.94*	0.09	*0.46*	*0.93*	0.03	*0.45*	*0.90*	0.02	*0.40*
Fattening (4weeks)	*0.93*	0.12	*0.53*	*0.93*	0.03	*0.53*	*0.91*	0.01	*0.54*
Fattening (8weeks)	*0.93*	0.09	0.06	*0.93*	0.03	0.06	*0.91*	0.00	0.09

### Logistic regressions

#### On-farm lifetime limb health index model

**Fig. 2:**
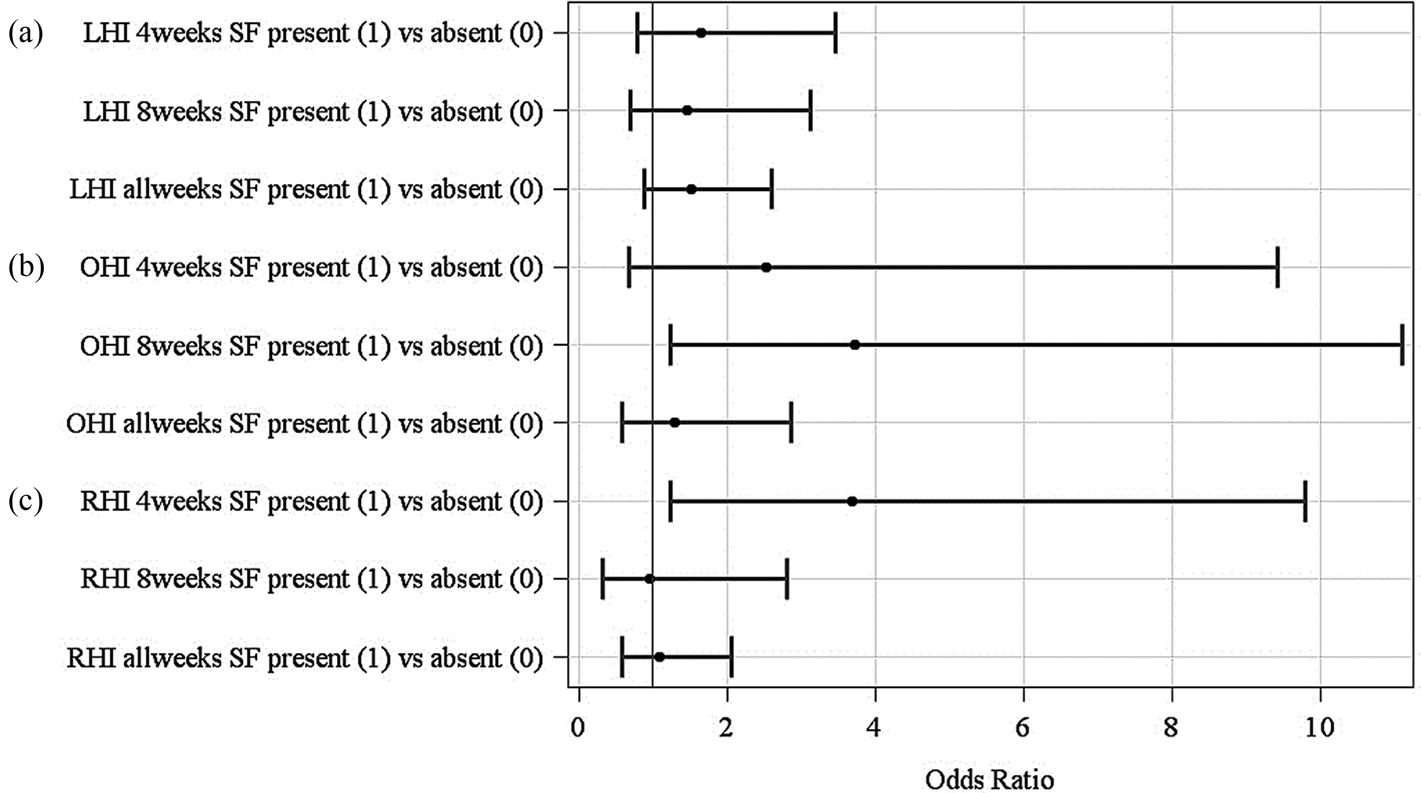
Odds ratios for the variables farm (A, B, C) and slaughterhouse findings (SF) (1 = present; 0 = absent) in the on-farm **(a)** lifetime limb health index (LHI) model, **(b)** lifetime other organ health index (OHI) model and **(c)** lifetime respiratory health index (RHI) model considering four (4weeks) or eight (8weeks) weekly assessments before slaughtering or considering all weekly assessments (allweeks) of the pigs’ lifetime

**Fig. 3:**
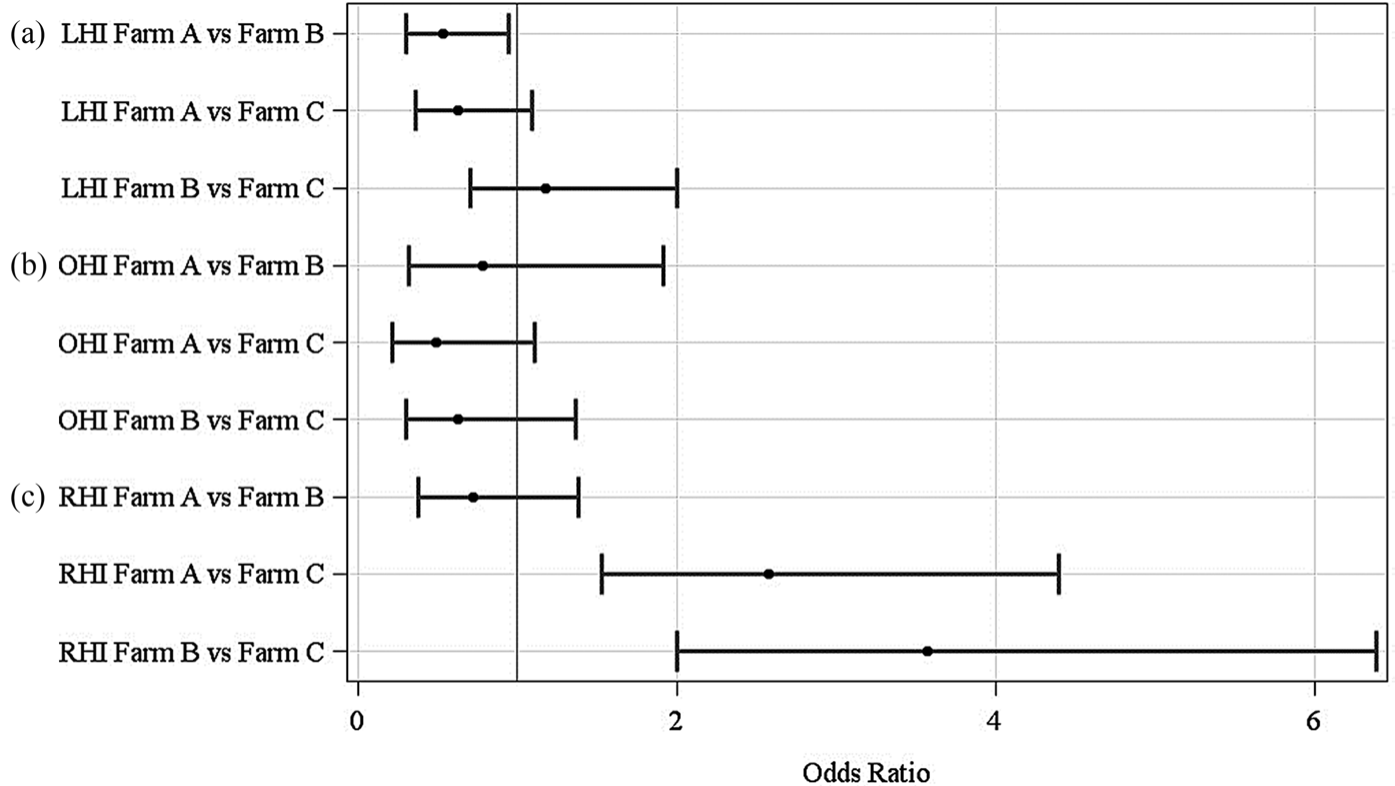
Odds ratios for the variable farm (A, B, C) in the on-farm **(a)** lifetime limb health index (LHI) model, **(b)** lifetime other organ health index (OHI) model and **(c)** lifetime respiratory health index (RHI) model considering all weekly assessments of the pigs’ lifetime

**Fig. 4:**
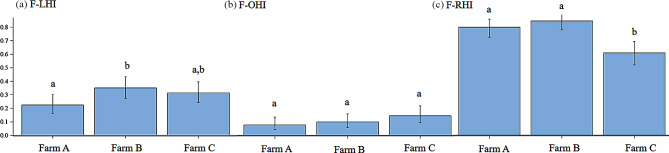
Least square means (LSM) and standard error of the farms for the on-farm **(a)** lifetime limb health index model (F-LHI), **(b)** lifetime other organ health index model (F-OHI) and **(c)** lifetime respiratory health index model (F-RHI). a, b significant differences between the farms (*P* < 0.05)

The results of the logistic regression model calculated a significant effect of the farm (*P* = 0.03) but not for the slaughterhouse findings on the F-LHI regardless of whether all weekly observations (allweeks), only the last eight (8weeks) or four (4weeks) weekly observations before slaughter are included in the logistic regression (see Fig. 2). When considering the odds ratios (OR) in the F-LHI-model, shown in Fig. 3, the lower risk of having a present (= 1) F-LHI on farm A (OR = 0.53; 95%-CI: 0.30 to 0.95) compared to farm B can be recognized provided that all weekly assessments (allweeks) are taken into account. For the other comparisons, no statistically significant difference could be determined. These calculations were also confirmed by the least square means (LSM) of this model (Fig. 4a), whereas the LSM of farm B (0.35; *P* = 0.03) is significantly different from the LSM of farm A (0.22; *P* = 0.13), but not to farm C (0.31; *P* = 1.00), again considering all weekly assessments (allweeks).

#### On-farm lifetime other organ health index model

For the F-OHI model, a significant effect of the slaughterhouse findings could be obtained considering the weekly assessments eight weeks (OR = 3.72; 95%-CI: 1.25 to 11.1) before slaughtering, but not for four weeks before slaughtering or if all weekly assessments were considered (see Fig. 2). For the effect farm no significant result was achieved considering all weekly assessments (allweeks). Thus, the probability of a present (= 1) F-OHI is not related to the slaughterhouse findings or farms either (see Fig. 3). The LSM of farm A is 0.08, of farm B 0.10, and farm C 0.14, but showed no significant differences (see Fig. 4b).

#### On-farm lifetime respiratory health index model

Examining the effect of slaughterhouse findings on the F-RHI, a significant effect could only be determined when the assessments four weeks (OR = 3.68; 95%-CI: 1.24 to 9.80) before slaughter were taken into account (not when the assessment eight weeks before slaughter or all assessments were considered), presented in Fig. 2. A significant effect of the farm could be found for the F-RHI model considering all weekly assessments (all weeks). As noticeable in Fig. 3, there was a higher risk for farm A (OR = 2.58; 95%-CI: 1.53 to 4.40) and for farm B (OR = 3.57; 95%-CI: 2.00 to 6.39) of a present F-RHI compared to farm C. In Fig. 4c, it was visible that the risk for having a present F-RHI was significantly lower for farm C (LSM = 0.61) compared to farm A (LSM = 0.80) and farm B (LSM = 0.85).

## Discussion

### Prevalence on-farm/slaughterhouse indicators

Of the twelve selected on-farm indicators, eight showed a prevalence below 5%. Other authors have also pointed out a low prevalence for animal-based indicators and, consequently, recommended evaluating farms with different husbandry systems to increase the variance of on-farm indicators [15]. This suggestion was followed in the present study, but the farms participated voluntarily and were well managed, which might have resulted in an above-average welfare. Besides the number of farms in the present study remained small, which limited the interpretation of the results. Nevertheless, the prevalence for most of the indicators was in a similar range as in previous studies. While bursitis was significantly higher in conventional farms studied by Maisano et al. [7] and Rocha et al. [16] with 38.0%, lower prevalence for hernia and lameness was found. The differences occurred probably on the one side because of the husbandry system of farm A (organic) in the present study, where no bursitis occurred due to the littered pens. On the other side, Maisano et al. [7] assessed heavy pigs one week before slaughtering, which increased risk of bursitis. The higher prevalence of hernias and lameness in the present study can be explained by the fact that the animals were observed on a weekly rhythm throughout their lives and thus also hernias or lameness at earlier ages were recorded (hernias can sometimes be wrongly identified at young age), which increases the prevalence, although the findings may have partially regressed.

Compared with the literature, in the present study the prevalence of the slaughterhouse indicators was in a lower range, especially for pleuritis (1.44%), pneumonia (5.28%) and bursitis (0.48%) [7, 17]. However, there is no international standardization in the collection of slaughterhouse indicators, which makes comparability difficult [12]. Therefore, both on-farm and slaughterhouse indicators have the general problem of multiple influences, such as the observers, the farm/slaughterhouse, the time of year and the lack of standardization, which can affect the reliability [15, 18].

### Data aggregation for the creation of the health indices

According to Fraser [19] animal welfare is a multidimensional concept, which requires multiple indicators to measure all welfare dimensions (biological functionality; natural living; affective state). However, these indicators have to be aggregated to obtain an overall statement about the animal welfare status of a farm [20]. For this reason, both the on-farm indicators and the slaughterhouse indicators were combined into indices according to disease complexes following the QS animal health index [21]. The use of the logical OR-operator takes into account that no balancing of present and absent welfare problems can occur [20]. Due to the aggregation of the on-farm data, the entire lifetime of the pigs was taken into account, which probably explains the higher numbers of affected animals in the on-farm indices compared to the slaughterhouse indices. However, it is important to keep in mind that no weighting was done in terms of timing and duration for the on-farm indicators. This means that an animal that was lame once in farrowing age was weighted in the same way in the further calculations as an animal that showed lameness and deviant behavior for the last two assessments before slaughter. Although this results in a loss of information and some relationships may be masked, this aggregation was considered necessary for further calculations, since it is known that some on-farm indicators had low prevalence and for the calculations of the agreement parameters, prevalence has an influence [15, 22]. In order to nevertheless be able to include a temporal aspect, the calculations were either additionally calculated for each production stage (agreement parameters) or taking into account a short time span (four or eight weeks) before slaughter (agreement parameters / logit regressions).

### Agreement parameters

Since exactly the same pigs were evaluated on-farm and at the slaughterhouse, a good agreement between the on-farm and slaughterhouse indices would be expected. This would allow the use of slaughterhouse indicators instead of on-farm indicators to measure some dimensions of on-farm animal welfare at the slaughterhouse. As advised in literature, different agreement parameters were calculated because each parameter has its own strengths and weaknesses [23]. When considering the agreement results, the high values (acceptable/good agreement) for the not chance-corrected PA were noticeable. In contrast, *k* is a chance-corrected measure of agreement and showed a low agreement overall for the three health indices. According to Byrt et al. [22] however, PA and *k* are influenced by the distribution of the prevalence of the data. Therefore, another kappa coefficient, the PABAK, was calculated, which was adjusted against the decrease in value due to an unbalanced prevalence [22]. Considering only the PABAK, all three indices (LHI; OHI, RHI) have in common that the agreement is acceptable to good for the farrowing and fattening period, but not for the rearing period. The reason could be increased fighting to establish rank order in the newly formed groups at the beginning of the rearing period [24]. Additionally, it is well known that weaning is one of the most stressful events in the pig’s life and can contribute to a weak immune system that results in reduced pig health [25]. Therefore, more short-term welfare issues (1 = present) were assessed for the pigs during this period, such as for lameness, back posture, deviant behavior, etc. The similar agreement between farrowing and fattening is unexpected, as it would imply that slaughterhouse indicators can be used to evaluate animal welfare on-farm in both the farrowing and fattening production period. However, one reason of the high agreement could be the low prevalence of the indicators mentioned above. Nevertheless, it could also be an indication that animals that have health issues in the farrowing unit, remain more vulnerable throughout their lives and thus pigs are more likely to have slaughterhouse findings [26]. This assumption is confirmed by Görge et al. [27], in which pigs with treatments in the rearing period had a higher probability of recording slaughterhouse findings. Therefore, the acceptable agreement for the RHI indicates the slaughterhouse indicators pneumonia, pericarditis, pleuritis as possible estimators of fever and deviant behavior, which are associated with pneumonia on-farm. Whereby, no assumption could be made for the on-farm indicators pumping and neurological problems because again of the low prevalence. The same can be said for mostly all OHI on-farm indicators (abdominal wall, body condition, rectal prolapse). The slaughterhouse indicators of the LHI (bursitis and joint inflammation) could be possible estimators of lameness on-farm. However, the S-LHI indicators probably do not predict claw alterations on-farm, because the claws had been cut off at the point of assessment in the slaughterhouse B. These statements can at least be made if you consider the results over the individual production stages. However, if the focus is shifted to the last weeks before slaughter (four or eight weeks before), it is evident that timing plays a decisive role in the agreement between on-farm assessments and slaughterhouse indicators.

A similar approach to which extent welfare on-farm can be measured retrospectively at the slaughterhouse was explored by Carroll et al. [28]. The results show that tail and skin lesions, acquired at least ten weeks before slaughter, could be identified at the carcass. In contrast, on-farm recorded health issues could not be detected at the carcass, but the pigs at slaughter were assessed for skin lesions, tail lesions, tail length and loin bruise. Therefore, they compared on-farm indicators with different indicators at the slaughterhouse, whereas in the present study, the equivalent health indices were compared, which explains the acceptable agreement between the on-farm and abattoir indicators at least four weeks before slaughter. However, according to the study by Carroll et al. [28], slaughterhouse indicators that record the exterior condition of the carcass (skin lesions, tail lesions, bursitis etc.) and less the inner organs of the pigs may be more suitable for the estimation of the welfare on-farm. They can be more easily detected at the slaughterhouse after scalding and dehairing of the carcass [8] than in the barns with moving animals. However, the inspection location is also critical at the slaughterhouse. For example, Harley et al. [8] suspects a different prevalence of tail lesions due to different inspection locations. Likewise, Keeling et al. [29] expresses concerns about mechanical damage to tails, which also occurred in the present study. It was not possible to determine whether the tails were intact, shortened or had lesions before the mechanical process (inspection after carcass cutting). Additionally, skin and ear lesions could not be used for the evaluations because of mechanical damages to the carcass, especially at slaughterhouse A. Hence, the fourth index ‘Integrity of the carcass’ could not be formed. Likewise, it was problematic to detect bursitis in the cold room due to the close confinement, onward transport and the more distant hindlimbs (on partially twisted carcasses). Also in the studies of Harley et al. [8] and Bottacini et al. [30] either only the hindlimbs or the forelimbs could be examined for bursitis.

The results mentioned above show the potential of slaughterhouse indicators to substitute on-farm indicators, provided that the inspection can be carried out at a suitable position, but largely they allow statements about one (biological functionality) of the three welfare dimensions. In addition, it must be ensured that the indicators used can distinguish between acute problems (which may arise during loading, during transport or in the holding pen) and chronic problems (which have arisen during the on-farm period) [31].

### Logistic regressions

Different effects of slaughterhouse findings on the three on-farm lifetime health indices (F-LHI, F-OHI, F-RHI) could not be confirmed using logistic regressions, whereas an effect of the farm could be determined for the F-LHI and F-RHI, considering all weekly assessments during the pig’s lifetime. The reasons for no effect of the F-RHI by the slaughterhouse findings could be due to the selection of on-farm indicators. For example, pumping did not occur at all, whereas fever was very frequent and could also be caused by other diseases. Also, Grosse-Kleimann et al. [32] found no relevant significant correlations between a respiratory score and on-farm indicators. It was expected that some health complexes, such as respiratory diseases, could be better assessed retrospectively using slaughterhouse indicators, compared to the broader other organ complex. This assumption was confirmed if only the assessments of the last four weeks before slaughter were included in the calculation. However, this influence could not be determined for the F-LHI, although very similar (on-farm: lameness; slaughterhouse: joint inflammation) or identical (bursitis) indicators were used on-farm and at the slaughterhouse. This may be due to the fact that not every lameness in the barn is due to joint inflammation and that it is only a snapshot and bursitis on-farm is difficult to assess in poor lighting conditions and with moving pigs [18]. The significant influence of slaughterhouse findings on the F-OHI eight weeks, but not four weeks before slaughter is considered unusual, as the slaughterhouse indicators (intestinal changes, liver lesions) are usually not visible on-farm [32].

So far, only a few studies have investigated the retrospective approach (and not use on-farm prevalence to predict slaughterhouse outcomes). Pessoa et al. [33] investigated predictions of slaughterhouse findings by on-farm indicators. They found that ear lesions have a high impact on the prediction of pneumonia, pleuritis and pericarditis at the slaughter line, which also confirms the assumption that certain disease complexes can be predicted better than others. Due to inadequate data indicators, describing the exterior condition of the carcass (except bursitis), could not be used for the calculation in the present study.

A major issue in predicting animal welfare on-farm based on slaughterhouse findings is the changing prevalence of on-farm indicators at different ages, which might have hidden possible relationships in the present study. For example, Witt et al. [34] determined that the indicators recommended for fattening pigs are not suitable for rearing piglets because they rarely occurred in this age group (e.g. bursitis, rectal prolapse). In contrast, other indicators occur more often in rearing piglets due to rank fights (lameness, back posture). This problem is also confirmed by Grosse-Kleimann et al. [32], where bursitis and lameness occurred more in older pigs and skin lesions as well as ear lesions more in younger pigs.

In addition to the difficulties which appear on-farm it is also already known that the meat inspection data were influenced by factors such as the farm, the meat inspectors, the season and the slaughterhouse [35]. The prevalence for liver lesions was highest for farm A. This may be due to the organic husbandry system because other authors also found more milk spots in animals from organic husbandry systems than in animals from conventional husbandry systems [36]. However, the influence of the slaughterhouse must be taken into account. The influence of the slaughterhouse on the variance of the prevalence of slaughterhouse indicators was confirmed by Klinger et al. [37]. The producer (farm) had the greatest influence with R^2^ = 0.61. This statement is also reflected in the results of the present study, where a significant effect of the farm was determined. The OR for a present F-LHI was higher on farm B compared to farm A and C. This may have occurred on the one side due to the frequently newly formed groups (rank fights) or on the other side due to the wide gaps to the outer walls of the fattening pens, in which the animals got sometimes stuck with a limb. This resulted in slight lameness. The higher probability of a present F-RHI was higher on farm A and B than on farm C. This may have been on the one side due to the rather high ammonia levels on farm B or a respiratory infection. On farm A, on the other side, the rectal temperature indicator probably had a strong influence. The pigs were allowed to walk down the aisle to measure their temperature during the fattening period and thus more movement occurred before the measurement. In addition, they were fed a lot of roughage, which also allows the core body temperature to rise because of the heat production of the microbial fermentation in the hindgut [38].

### Conclusion

In the present study, three on-farm and three slaughterhouse health indices (limb health, other organ health, respiratory health) of pigs were defined. Acceptable agreement for the farrowing and fattening period between on-farm and slaughterhouse were found for the respiratory and the limb health indices, but not in the rearing period. This indicates that on-farm welfare can be explained to a certain extent by meat inspection data. This applies in particular to the last four weeks before slaughter. For example, the slaughterhouse indicators pneumonia, pericarditis and pleuritis can predict fever and deviant behavior (signs of pneumonia) on the farm. Likewise, the slaughterhouse indicators bursitis and joint inflammation are suitable to detect lameness problems on farms. Although some assumptions regarding time variations can be made, it could not be conclusively clarified how long on-farm welfare can be explained retrospectively with the use of meat inspection data. This is due, on the one side, to the often low and nonetheless changing prevalence of the on-farm indicators depending on the age class and, on the other side, timing and location of the inspection at the slaughterhouse is important for reliable assessments. Future studies with a higher number of farms are needed to increase the variance of the welfare indicators. Besides, the seemingly suitable indicators allowed statements about only one (biological functionality) of the three welfare dimensions.

## Methods

### Data collection

The data collection was carried out between October 2020 to February 2022 on three voluntarily participating farms with a closed system in Schleswig-Holstein, northern Germany. These farms differ in their husbandry systems (farm A: 50 sows, organic; farm B: 1400 sows, conventional; farm C: 60 sows, conventional) and in their production factors. All male piglets were castrated and the piglets of farms A and C had undocked tails. Those of farm B had docked tails. More details of the farms can be found in Witt et al. [39].

The duration of the data collection allowed an on-farm assessment of pigs from three batches (batch = time period from birth to slaughter) per farm, with the exception of farm C, on which the pigs of four batches were considered to reach a similar number of assessed pigs (see Table 2). The trial started with the random selection of a group of sows to be farrowed, ranging from 4 to 10 sows depending on the herd size of the three farms. In the week after farrowing, the first assessment of the animals in this study, i.e. the piglets of the randomly selected sows, took place. The piglets on all farms were individually marked due to management reasons, which allowed a weekly individual welfare assessment of each pig from birth until slaughter. As a part of routine health checks the pigs were restrained and the weekly assessments by the same observer were performed simultaneously. Due to the different husbandry systems and barn capacities on the three farms, the individual residence times of the pigs in the different production stages varied, but in all three farms the pigs were transferred to the fattening barns with a body mass of approximately 30 kg. The pigs were slaughtered with a body mass of approximately 120 kg. The number of pigs that were part of this study depended on the number of piglets born alive from the group of sows (40 to 120 piglets per batch) and on which of these animals could be identified at slaughterhouse. In total 628 pigs were assessed both on-farm and at slaughterhouse and were included in the evaluations of this study.

**Table 2:** Overview of the batches and included animals of the three farms in the study (n = numbers)

Farm	A	B	C
Production type	Organic	Conventional	Conventional
n batches	3	3	4
n pigs	198	188	242
Slaughterhouse	A	B	B

Due to individual growth differences, several slaughter dates were carried out per batch, resulting in different numbers of weekly assessments of the 628 pigs. On average, the number of weekly assessments per pig was 24 (21–30).

### On-farm indicators

The weekly assessments of the individually marked pigs were carried out by one observer, who had been trained in the assessment of all on-farm welfare indicators used. The weekly assessment of the pigs was based on selected health and welfare indicators from different protocols that had been recommended with regard to use in regular self-assessment to fulfill the national law requirement. In particular, the indicators included were derived from the Welfare Quality protocol for pigs [4], the German guideline for farm self-monitoring [40] and standard health checks from veterinary routine practice [41]. The resulting individual protocol for this study contained 12 animal-based, individual-level, on-farm indicators. The complete list of the on-farm indicators, their source, their definitions and scoring scale are presented in Table 3. There are indicators with a three-point scale (category 0 = absent, category 1 = light appearance, category 2 = strong appearance) and indicators with a two-point scale (category 0 = absent, category 1 = present). All individually marked pigs were scored with the on-farm indicators following the guidelines and explanations of the respective protocols or descriptions they originated from. Both body sides were considered to detect all health and welfare issues.

**Table 3:** Animal-based, individual-level, on-farm indicators with category, source, scoring scale and definition. Only those categories indicating presence of a welfare issue (categories 1 and 2) are shown. (KTBL = German guideline for self-monitoring [40]; Vet = standard veterinarian health check [41]; WQP = Welfare Quality protocol for pigs [4])

Indicator	Source	Category	Definition
Abdominal wall^*^	Vet	1	Abnormal (tense)
Back posture	Vet	1	Abnormal (arched back)
Body condition	WQP	1	Thin: visible spine, hip, pin bones
Bursitis^*^	WQP	1	One/several small bursae on the same leg or one large bursa
		2	Several large bursae on the same leg or one extremely large or eroded bursa
Claw alterations^*^	KTBL	1	Evidence of alterations (injured, bleeding erosion (side wall), cracks (heel, sole, sole/heel junction, side wall), panaritium)
Fever	Vet	1	Rectal temperature > 40 °C
Lameness^*^	WQP	1	Severely lame, weight-bearing on affected limb
		2	No weight-bearing on one limb or unable to walk
Neurological disorders	WQP	1	Evidence of neurological problem (head tilt)
Normal behavior	Vet	1	Abnormal behavior (lethargic)
Pumping	WQP	1	Evidence of labored breathing
Rectal prolapse	WQP	1	Evidence of rectal prolapse
Ruptures/hernias^*^	WQP	1	Small hernia/rupture
		2	Hernia/rupture touching the floor or with bleeding lesion

^*both sides of the pigs were considered for the assessment^

### Slaughterhouse indicators

In addition to the on-farm assessments, the individually marked pigs were evaluated at the two slaughterhouses (see Table 2) by the official veterinarian according to the routine veterinary meat inspection in Germany. The meat inspection followed the General Administrative Regulation for Food Hygiene (AVV LmH) and is required by the Regulation (EU) 2019/627 [9]. The definitions of the seven slaughterhouse indicators used for the evaluation in this study are presented in Table 4 and based on General Administrative Regulation for Food Hygiene (AVV LmH) [42].

**Table 4:** Slaughterhouse indicators with category, scoring scale and definition based on General Administrative Regulation for Food Hygiene (AVV LmH). Only those categories indicating presence of a welfare issue (categories 1, 2 and 3) are shown [42]

Indicator		Grade	Definition
Bursitis	1		Bursa > 5 cm diameter present
Intestinal changes	1		Inflammations present
Joint inflammation	1		Inflammations present
Liver lesions	1		Milkspot/s present
Pericarditis	1		Altered
Pleuritis	1		< 0% - ≤10% affected by pleuritis
	2		< 10% - ≤30% affected by pleurits
	3		< 30% affected by pleuritis
Pneumonia	1		< 0% - ≤10% affected by pneumonia
	2		< 10% - ≤30% affected by pneumonia
	3		< 30% affected by pneumonia

### Defining lifetime health indices on-farm

To examine the agreement between on-farm and slaughterhouse indicators, data were first aggregated (Fig. 1). For this purpose, the three-point scale on-farm indicators were combined into a two-point scale, whereby the categories 1 (light appearance) and 2 (strong appearance) were combined into 1 (present) for every weekly assessment in a weekly score of each indicator (WS) of each pig. To sum up all weekly scores of an indicator into one binary lifetime score (LS) of an indicator per pig, 0 (absent) was assigned if all WS of a pig were rated 0 (absent) and assigned a 1 (present/welfare issue) if at least one WS was rated 1 (present). In the next step, the on-farm indicators (LS) were assigned to disease complexes, combining them into the following on-farm lifetime health indices, similar to QS animal health indices [21] for meat inspection data (see Fig. 1):

1) Limb health index (F-LHI), assigned indicators were back posture, bursitis, claw alterations, lameness.

2) Other organ health index (F-OHI), assigned indicators were abdominal wall, body condition, rectal prolapse, ruptures/hernias.

3) Respiratory health index (F-RHI), assigned indicators were deviant behavior, fever, neurological disorders, pumping.

A pig was scored a 0 (absent) in a lifetime health index if all four assigned indicators were scored 0 in the LS. If at least one of the assigned indicators was scored 1 in the LS, the lifetime health index was scored 1 as well. Due to the calculation using the logical OR-operator, the lifetime health index score can only be 0 (absent) or 1 (present/welfare issue), even if a pig had been scored (LS) 1 (present/welfare issue) in all four assigned on-farm indicators for this index.

### Defining health indices at slaughter

For the slaughterhouse indicators, the categories 0 (not altered) and 1 (0-10%) were combined into 0 (= absent) and the categories 2 (10-30% altered) and 3 (> 30% altered) were combined into 1 (= present). This results in binary data for all slaughterhouse indicators. The three disease complexes mentioned above were also used to generate combined indices with the slaughterhouse indicators in the same manner for each pig (see Fig. 1). For this purpose, the slaughterhouse indicators are also assigned to the corresponding indices according to the specifications of the QS animal health index [21]:

1) Limb health index (S-LHI), assigned indicators were bursitis, joint inflammation.

2) Other organ health index (S-OHI), intestinal changes, liver lesions.

3) Respiratory health index (S-RHI), pericarditis, pleuritis, pneumonia.

The calculation of the slaughterhouse indices for each pig was performed, as explained above, with the use of the logical OR-operator and resulted in a binary health index score for each pig. Due to missing slaughterhouse data (skin, ear or tail lesions; drift marks), the fourth health index of the QS animal health index ‘integrity of the carcass’, could not be calculated. Neither was a corresponding on-farm index created. For later analysis (logistic regressions) the three slaughterhouse indices (S-LHI, S-OHI, S-RHI) were combined into the variable slaughterhouse findings using an OR-operator. Pigs with at least one present (1) slaughterhouse indicator were therefore rated 1 (present) in the slaughterhouse findings.

### Statistical analysis

To present the distribution of the binary scores per health index, the respective number of animals affected with a welfare issue (index scored with 1) per health index were determined and the confidence intervals were calculated with the statistical software SAS 9.4 [43]. The first part of the analysis determined the agreement between the on-farm and slaughterhouse health indices at individual animal-level, taking the production period and additionally four or eight weeks before slaughtering into account, by using different agreement parameters, described below. The second part of the analysis investigated the relation of slaughterhouse findings and the individual on-farm lifetime health indices by using logistic regressions to determine whether these had a particularly strong influence on individual on-farm indices. For the calculations of the effect of the slaughterhouse findings, either all weekly assessments (allweeks) or only the assessments eight (8weeks) or four (4weeks) weeks before slaughter were taken into account.

### Agreement parameters

#### Percent agreement

The PA expresses the agreement of the units as a percentage of the total number of units. A value of 100% describes perfect agreement und a value of 0.00% no agreement. Since it is a descriptive test, which has no predictive power, the thresholds for an acceptable and good agreement between the measurements must be determined separately for each study [44, 45]. In this study, a deviation of ≥ 90.0% was considered as acceptable and a deviation of ≥ 95.0% was considered as good agreement in accordance with de Vet et al. [23].

#### Cohen’s kappa coefficient

The *k* is a useful chance-corrected measure for quantifying agreement of dichotomous judgments. It is calculated with the ratio between the occurred proportion of agreement between the measurements and the maximum possible proportion of agreement. Negative values (up to -1.00) indicate less agreement than expected from chance alone, positive values (up to 1.00) represent more agreement than expected from chance [45, 46]. Values of ≥ 0.40 were interpreted as acceptable and values of ≥ 0.60 as good agreement [47].

#### Prevalence-adjusted bias-adjusted kappa

The PABAK is adjusted for bias and prevalence by Byrt et al. [22]. Acceptable agreement is assumed for values ≥ 0.40 and good agreement for values ≥ 0.60 according to Plesch et al. [48].

### Logistic regressions

Logistic regressions were used to estimate the effects of slaughterhouse findings in general on on-farm lifetime health indices at animal-level. Additionally, the assessments eight (8weeks) and four (4weeks) weeks before slaughter were considered to determine the retrospective impact of the slaughterhouse findings on the on-farm health indices. For this purpose, the regressions were created step by step and selected using the Akaike’s information criteria (AIC) and the deviance divided by its degrees of freedom (target value = 1). The respective on-farm lifetime health indices (F-LHI, F-OHI, F-RHI) were selected as the dependent binary variable. The final regressions models for the F-LHI, F-OHI and F-RHI, respectively, included the fixed effects farm (1, 2, 3), and slaughterhouse findings (0, 1). The interaction between farm and slaughterhouse findings was tested but no significant influence was calculated, and no improvement of the model quality was achieved. The statistical significance value was set at *P* < 0.05. The OR, LSM and their standard errors were considered for interpretation.

## Data Availability

All data generated or analyzed during this study are included in this published article. Original data sets are available from the corresponding author upon reasonable request.

## Abbreviations

CI: Confidence intervals
EU: European Union
F-LHI: On-farm limb lifetime health index
F-OHI: On-farm other organs lifetime health index
F-RHI: On-farm respiratory lifetime health index
*k*: Cohen's Kappa Coefficient
LS: Lifetime score of each indicator
LSM: Least square means
OR: Odds ratio
PA: Percent agreement
PABAK: Prevalence-Adjusted Bias-Adjusted Kappa
S-LHI: Slaughterhouse limb health index
S-OHI: Slaughterhouse other organs health index
S-RHI: Slaughterhouse respiratory health index
WS: Weekly score of each indicator
